# Impact of Income on Small Area Low Birth Weight Incidence Using Multiscale Models

**DOI:** 10.3934/publichealth.2015.4.667

**Published:** 2015-10-10

**Authors:** Mehreteab Aregay, Andrew B. Lawson, Christel Faes, Russell S. Kirby, Rachel Carroll, Kevin Watjou

**Affiliations:** 1 Department of Public Heath Sciences, Division of Biostatistics and Bioinformatics, MUSC, Charleston, USA; 2 Interuniversity Institute for Biostatistics and statistical Bioinformatics, Hasselt University, Hasselt, Belgium; 3 Department of Community and Family Health, University of South Florida, Tampa, FL, USA

**Keywords:** Low birth weight (LBW), predictive accuracy, shared multiscale model, independent multiscale model, scaling effect, Bayesian multiscale model

## Abstract

Low birth weight (LBW) is an important public health issue in the US as well as worldwide. The two main causes of LBW are premature birth and fetal growth restriction. Socio-economic status, as measured by family income has been correlated with LBW incidence at both the individual and population levels. In this paper, we investigate the impact of household income on LBW incidence at different geographical levels. To show this, we choose to examine LBW incidences collected from the state of Georgia, in the US, at both the county and public health (PH) district. The data at the PH district are an aggregation of the data at the county level nested within the PH district. A spatial scaling effect is induced during data aggregation from the county to the PH level. To address the scaling effect issue, we applied a shared multiscale model that jointly models the data at two levels via a shared correlated random effect. To assess the benefit of using the shared multiscale model, we compare it with an independent multiscale model which ignores the scale effect. Applying the shared multiscale model for the Georgia LBW incidence, we have found that income has a negative impact at both the county and PH levels. On the other hand, the independent multiscale model shows that income has a negative impact only at the county level. Hence, if the scale effect is not properly accommodated in the model, a different interpretation of the findings could result.

## 1 Introduction

Low birth weight (LBW) is an important public health issue around the globe. The term low birth weight is used to describe babies who are born weighing less than 2500 g [1]. These are further subdivided into very low birth weight (VLBW), which is less than 1500 g and extremely low birth weight (ELBW), which is less than 1000 g [2]. In 2011, a study in the US found that of the 3.8 million births observed, 6.1% were diagnosed with LBW, whereas 1.3% were diagnosed with VLBW [3]. While the majority of low birth weight infants have normal outcomes, as a group they generally have higher rates of subnormal growth, illness, and neurodevelopmental problems which increases as the newborn's birth weight decreases [4]. At the individual level, LBW is an important factor associated with higher risks of infant and childhood mortality [5]. In addition, at the population level, the proportion of LBW births is an indicator of public-health problems.

The primary causes of low birth weight are premature birth and fetal growth restriction, usually due to infection and birth defects. In general, the risk factors associated with low and very low birth weight are maternal smoking, multiple births, maternal or fetal stress, prenatal alcohol and drug use, poor prenatal nutrition, and violence toward the pregnant woman [6]. There are some studies which show that LBW declines as the socioeconomic status increases [7]. While many studies suggest that individual-level risk factors play a significant role in explaining LBW outcome, little research exists examining the geography of important explanatory factors related to the incidence of LBW. For county-level counts of LBW in Georgia and South Carolina, Kirby et al [8] found that income is a negative risk factor while the proportion of black population is a positive risk factor for the number of LBW. In this paper, we aim to investigate whether or not income is negatively associated with LBW at different scale levels simultaneously. To achieve this goal, we apply the methods proposed by Aregay et al [9].

Recently, Aregay et al [9] have proposed statistical methods termed as *multiscale models* that are useful to address the scaling effect which exists in aggregated geographical data. A spatial scaling effect occurs when data are aggregated from a finer (smaller) to a coarser (larger) geographical level. This process results in smoothing out the variation at the finer scale level and hence, information will be lost during data aggregation. If the scaling effect is not accommodated, an erroneous conclusion may be drawn. For example, a risk factor which predicts the burden of LBW at the finer level may not provide a consistent result at the coarser level. We demonstrate this on the LBW outcome obtained from the Georgia state with 159 counties that are nested within 18 public health (PH) districts.

The paper is organized as follows. In Section 2, we present the data structure and the exploratory data analysis for the predictor and the outcome of interest. In Section 3, we describe the statistical methods which we apply to the data set. In Section 4, we provide the results obtained from the models fitted to the data. Finally, in Section 5, we discuss the results and draw conclusions.

## 2 Low Birth Weight in Georgia

**Figure 1.**
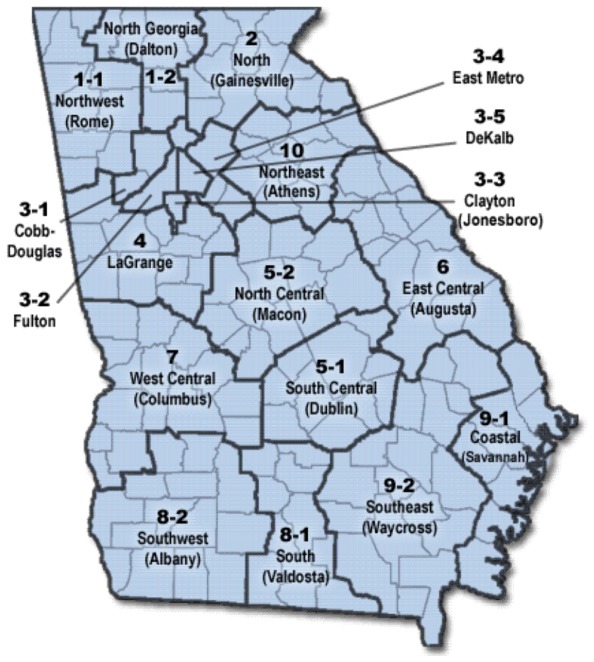
State of Georgia, USA: County and PH district boundary map.

To apply the methods which will be described in Section 3, we choose to examine low birth weight incidence in the counties of Georgia (GA). The Georgia state map was selected as it provides a reasonably large set of spatial units at each scale level and therefore a degree of spatial variation in disease risk could be found within the study area. The data are available at both counties and PH levels from the state of Georgia via the Georgia Division of Public Health OASIS system (http://oasis.state.da.us). In Georgia, there are 159 counties nested within 18 PH districts as shown in Figure 1. On average nine counties are nested within each public health district. The outcome of interest is the number of low birth weight (LBW) births while the predictor is median household income of the residents of a given county. The LBW count at the PH district level is obtained by summing up the LBW for the counties that are nested within the PH district, whereas the income predictor at the PH level is obtained by averaging the income of the counties within the PH district. The aim is to investigate the relationship between the predictor income and LBW at both the county and PH levels simultaneously. The predictor median household income is available through the Area Health Resources Files (AHRF) dataset. We have selected the recent data (both income and LBW incidence) in 2007.

**Figure 2.**
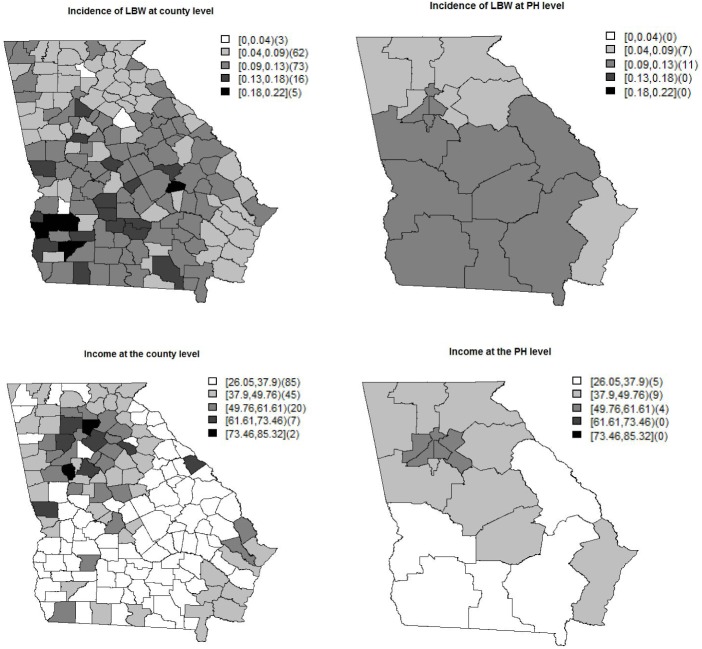
LBW at the county and PH levels (top figure) and median household income at the county and PH levels (bottom figure).

The exploratory data analysis for the LBW incidence and median household income at both the county and PH district is shown in Figure 2. As can be seen from the figure, there is an increased risk of having a LBW baby in southwest of Georgia at the county level, while the median income in those areas is relatively low. Similarly, there is a high incidence of LBW at the PH level in the southwest region of the map, and a relatively small LBW incident is present in the northern GA. On the other hand, a relatively high income is observed in the northern GA. This shows that income may have a negative impact on LBW incidence. From the figure, we can also clearly see that scaling up smooths out the variation for both LBW and income. Our analysis of these data with and without the scale effect is deferred to Section 4.

## 3 Multiscale Models

Multiscale models are used to describe data at different geographical levels. These methods allow us to study the relationship between predictors and outcome at multiple scales simultaneously. The methods can help us to investigate whether or not the relationship at the finer level will hold true at the coarser level. In this paper, it is of interest to apply multiscale models to model the relationship between spatially referenced outcomes and predictors by taking into account the spatial scaling effect. Suppose *Y_i_k__* is the outcome random variable for region *i*; *i* = 1; . . . ; *N_k_*, at scale level *k*. Here we focus on two level geographical data and hence *k*=1,2, *N_k_* is the number of regions at the scale level *k* with *k*=1 denoting the finest (county) level, *k*=2 the coarsest (PH) level. Further, assume that *X_i_k__* is the observed covariate for geographical area *i* at the *k^th^* scale level. For spatial health data, it has been recognized that a geographical correlation will exist between spatial units. This is due to the fact that spatial units close together in space often have similar disease risk, whereas regions far apart are often different. Hence, a spatially structured random effect is used to handle the association between neighbors.

Conditioning on the spatially structured random effect *v_i_k__*, we assume that the outcomes *Y_i_k__* are independent with densities $f\left(y_{i_{k}}|v_{i_{k}},{\text{β}}_{k};{\text{μ}}_{i_{k}}\right)$ with $h\left({\text{μ}}_{i_{k}}\right)={\text{β}}_{0_{k}}+{\text{β}}_{k}X_{i_{k}}+v_{i_{k}}+{\text{ε}}_{i_{k}}$ for a known link function *h*, such as, identity, logit, and log link for continuous, binomial, and count data, respectively. The unstructured random effects ${\text{ε}}_{i_{k}}$ are $N\left(0,\sigma_{{}_{\varepsilon_{k}}}^{2}\right)$ and $v_{i_{k}}$ is assumed to have an intrinsic conditional auto-regressive (ICAR) structure [10]. In this paper, we focus on modeling binomial data at different scales. For the purpose of comparison, we applied two models: the first model does not take into account the scaling effect, whereas the second model does so. For both models, we use a Bayesian approach that combines the current and previous information. The advantage of using the Bayesian paradigm is that it includes the uncertainty of the parameters via the prior distribution. Next, we describe the application of the models to investigate the impact of income on Georgia LBW incidence at both the county and PH levels simultaneously. To our knowledge, the application of the methods for such purpose is a novel work and have never been explored before.

### 3.1 Model 1

In this naive approach, we consider an independent multiscale convolution model which does not introduce linkage between the different scale levels. We have two scale levels in the state of Georgia: the county and PH levels. Thus, the independent multiscale model can be expressed as: 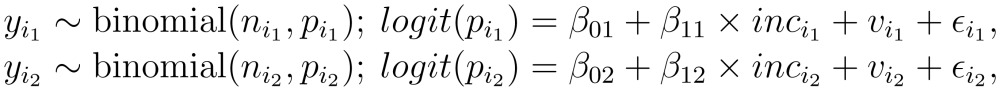
(1) where *y*_*i*_1__, *inc*_*i*_1__, and *n*_*i*_1__ are the number of LBW births, the median household income, the number of births at the county level and *y*_*i*_2__, *inc*_*i*_2__, and *n*_*i*_2__ are the total number of LBW, average of the median household income, and the total births of the counties nested within the PH level, respectively. Further, *v*_*i*_1__ and *v*_*i*_2__ are the correlated heterogeneity (CH) at the county and PH levels, whereas *ε*_*i*_1__ and *ε*_*i*_2__ are the uncorrelated heterogeneity (UH) at the county and PH levels. We assumed the following ICAR structure for the correlated random effects: 
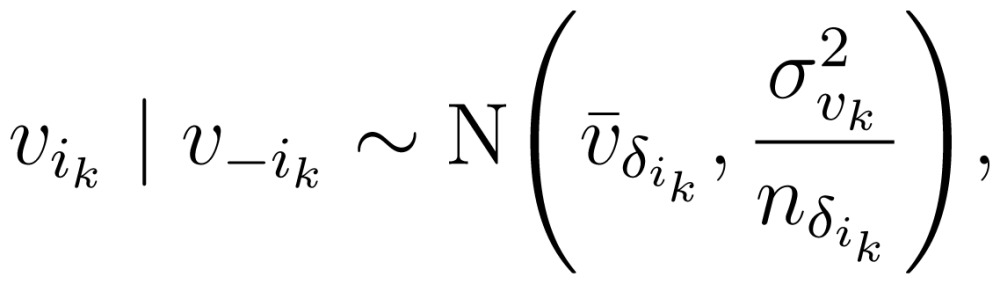
(2) where 
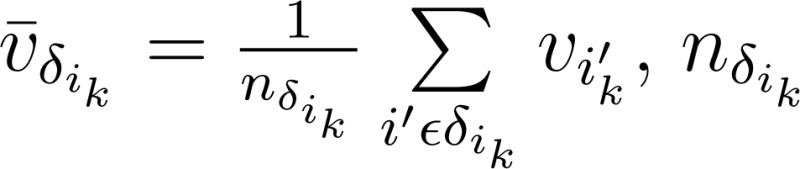
 is the set of labels of the neighbors of unit *i* at scale level *k*=1,2 and *v*_−*i*_*k*__ is the set of all random effects not including the *i^th^*. For the uncorrelated random effect, we assumed 
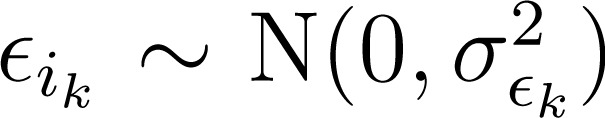
, where *k*=1,2. A uniform prior distribution was assumed for the standard deviation of the correlated and uncorrelated random effects, i.e., ${\text{σ}}_{v_{k}}\text{\textasciitilde{}U}\left(0,100\right)$ and ${\text{σ}}_{\varepsilon_{k}}\text{\textasciitilde{}U}\left(0,100\right)$ [11]. Further, we considered a flat prior for the intercepts *β*_01_ and *β*_02_, and a non-informative normal distribution for the slope parameters *β*_11_ and *β*_12_. Note that there is no linkage between the convolution models at the county and PH levels. Hence, this model does not address the scaling effect.

### 3.2 Model 2

In this section, we applied the shared multiscale model proposed by Aregay et al [9]. Their work involves the use of joint convolution models to link the different scale levels via a shared spatially structured random effect *v*_*i*_2__. The shared random effect was used to recover the lost information during data aggregation. Their model can be easily applied in standard software such as WinBUGS because it is based on convolution models that are widely used in spatial epidemiology. For the state of Georgia with two levels, the multiscale convolution model can be given as: 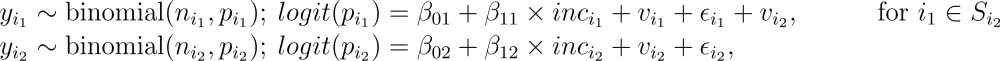
(3) where *S*_*i*_2__ is the set of subregions at the county level within the PH district. The shared random effect *v*_*i*_2__ is common for all the counties nested within the PH district. For example, if a given PH district has five counties, *v*_*i*_2__ will be the same for all the five counties. In other words, the counties share the characteristics that they inherit from their parent which is here the PH district. Note that we assumed the same prior distribution for all the model parameters as in Model 1.

### 3.3 Model assessment and goodness of fit

In the previous sections, we have described two models: while the first model ignores the scale effect, the second model includes a parameter that takes care of the effect. To asses the benefit of the second model, we need techniques which compare the models in terms of model fit as well as prediction accuracy. In this paper, we considered the deviance information criterion (DIC [12]–[13]) for model selection and the mean square prediction error (MSPE [14]) to assess the predictive ability of the models. DIC is composed of the deviance that explains the model fit and the number of effective parameters (PD) which penalizes for model complexity. The MSPE compares the predicted values with the observed values of the outcome and helps us to investigate the prediction accuracy of the model. In general, a model with smaller values of deviance, PD, DIC and MSPE is selected to be the better model as compared to the other model. A DIC difference between 3 and 7 is considered to be an improvement in the model performance [12].

## 4 Results

We fitted the models using Monte Carlo Markov Chain (MCMC) via the R2WinBUGS package. To improve convergence, the covariate income was standardized. We generated three chains of 30,000 Monte Carlo samples after discarding the first 15,000 start up samples. Hence, the posterior estimates are based on 45,000 Monte Carlo samples. The scale reduction factor ( was equal to one for all the parameters), BGR and trace plots [15] suggest that a reasonable convergence has been achieved for all the model parameters. The DIC and MSPE results using Models 1-2 are shown in Table 1. We can see that Model 2 improves the model fit at both the county and PH levels. At the same time, the number of effective parameters (PD) obtained from Model 2 is lower than those of Model 1. Moreover, the predictive ability of Model 2 is better than Model 1. Hence, overall, using the shared correlated random effect for scaling effect has a benefit as compared to the naive approach.

**Figure 3.**
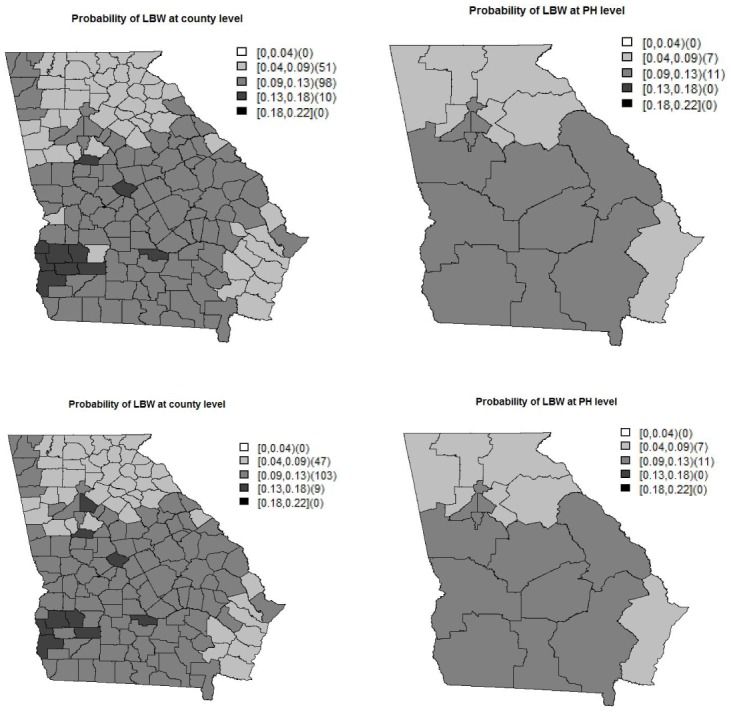
Probability of LBW outcome obtained from Model 1 (top figure) and probability of LBW obtained from Model 2 (bottom figure) at both the county and PH levels.

In contrast, applying the naive independent multiscale model may lead to wrong conclusions as can be seen in Table 2. Using Model 2, income has a negative impact on the incidence of LBW at both the county (*β*_11_) and PH (*β*_12_) levels. On the other hand, Model 1 provides inconsistent results between the county and PH levels; income has a negative impact at the county level but not at the PH level. This could be due to the lost information which is not recovered in Model 1. The probability of the risk of having a LBW baby at the different counties and PH districts are shown in Figure 3. The results are consistent with the results obtained from the exploratory data analysis in the top panel of Figure 2 seen in Section 2. As can be seen from the figure, there is high risk of increased incidence of LBW in the southwestern region of GA, which has a relatively lower median household income as compared to the other regions (see bottom panel of Figure 2). The difference between Figure 2 and Figure 3 is that the latter results in smoother risk estimates at the county level. This is due to the correlated component (*v*_*i*_1__) that smooths out the spatial variation between the counties.

The interpretation of the odds ratio, which measures the amount of relationship between income and LBW incidence, is as follows. Since income covariate in the model has been standardized, we have to transform back the parameter estimates to explicitly interpret in the original scale of the predictor income. To do this, we divide the slope parameter estimate by the standard deviation of the income covariate. For example, for the best model which is Model 2, the slope parameter estimate at the county level is −0.09, i.e., *β*_11_=−0.09 (see Table 2). The standard deviation of income is 10.659. The transformed estimate will be −0.09/10.659=−0.008. Moreover, To obtain the odds ratio estimate, we have to use the exponential function to the transformed estimate, i.e., odds ratio=exp(−0.008)=0.992. This result indicates that for every one thousand dollars increase in the median household income, we expect to see about 0.8% (1-0.992) decrease in the odds of having a LBW birth. Hence, counties with high income are less likely to have LBW births as compared to the counties with low income. Similarly, we can calculate the odds ratio at the PH level using the transformed estimates. The slope parameter estimate at the PH level (*β*_12_; see Table 2) is equal to −0.06 and the standard deviation of the income covariate at the PH level is 8.264. The odds ratio for the transformed estimate can be calculated as exp(−0.06/8.264)=0.993. Thus, at the PH level, for one thousand dollar increase in the median household income, we expect to see about 0.7% (1-0.993) decrease in the odds of having a LBW birth. Note that the odds ratio estimate at the PH district is similar to the odds ratio estimate at the county level.

**Table 1.** Model fit and predictive accuracy results for Georgia LBW data. Model 1 represents an independent multiscale model that ignores the scaling effect and Model 2 denotes a shared multiscale model which handles scaling effect.

Models	PD		DIC		MSPE	
	county	PH district	county	PH district	county	PH district
Model 1	75.64	17.29	1076.15	184.93	163.1	1439.0
Model 2	62.32	14.76	1072.63	181.35	162.5	1418.0

**Table 2.** Georgia LBW rate data. Posterior mean estimates and standard error. Model 1 represents an independent multiscale model that ignores the scaling effect and Model 2 denotes a shared multiscale model which handles scaling effect.

Models	Mean								95% CI							
	*β*_01_	*β*_02_	*β*_11_	*β*_12_	*σ*_*v*1_	*σ*_*ε*1_	*σ*_*v*2_	*σ*_*ε*2_	*β*_01_	*β*_02_	*β*_11_	*β*_12_	*σ*_*v*1_	*σ*_*ε*1_	*σ*_*v*2_	*σ*_*ε*2_
Model 1	-2.21	-2.24	-0.12	-0.01	0.24	0.10	0.28	0.09	(-2.25,-2.18)	(-2.30,-2.18)	(-0.16,-0.07)	(-0.13,0.10)	(0.15,0.35)	(0.04,0.16)	(0.05,0.48)	(0.001,0.23)
Model 2	-2.19	-2.24	-0.09	-0.06	0.06	0.12	0.32	0.04	(-2.23,-2.16)	(-2.27,-2.21)	(-0.13,-0.06)	(-0.12,-0.01)	(0.01,0.16)	(0.08,0.16)	(0.21,0.49)	(0.001,0.09)

## 5 Discussion and Conclusion

In this paper, we have applied the shared multiscale model proposed by Aregay et al [9] to examine the relationship between income and LBW incidence obtained from the state of Georgia with two levels: county and PH levels. For the purpose of comparison, we have also employed an independent multiscale model which does not take into account the scale effect. As such, we have found that using the shared multiscale model improves the model fit and predictive accuracy as compared to the independent multiscale model. In addition, the shared multiscale model provides consistent results at the county and PH levels, whereas the independent multiscale model produces inconsistent results between those levels. In particular, the shared multiscale model shows that income has a negative impact on LBW incidence at both the county and PH levels. At the county and PH level, for every one thousand dollars increase in the median household income, we expect to see about 0.8% and 0.7% decrease in the odds of having a LBW birth, respectively. On the other hand, using the independent multiscale model, we have found that income has a negative impact only at the county level.

In both developed and developing countries, LBW is an important cause of short-term morbidities such as respiratory distress syndrome and long-term morbidity like blindness, and mental retardation. This results in excessive medical cost to treat the infants. LBW can also cause childhood mortality. In the US, in 2009, 5.3% of Low birth weight infants died as compared to 0.2% normal birth weight infants [16]. In 2006, the rate of LBW was peaked to 8.26% and has decreased slightly since then [17]. Although there is a slight decline in the LBW rate globally, it is as high as 30% in many developing countries [18]. There are many important risk factors which play a significant role in the incidence of LBW.

The two main causes of LBW are premature birth and intrauterine growth restrictions (IUGR). In addition, there are risk factors related with LBW such as smoking, poor nutrition, stress, alcohol and drug use. There are also studies which show that LBW-income women who suffer from chronic psychological stress are at increased risk of having a LBW baby [19]. For the counties within the Georgia and South Carolina states, Kirby et al [8] have found that income has a negative impact on LBW rate. Our results are in agreement with the results obtained by Kirby et al [8] at the county level of the Georgia state. Moreover, we have calculated the LBW incidence at the PH level. Still, income predicts the LBW outcome at the PH district.

There are many strategies that can be adopted to reduce the LBW birth rate at both the individual and population levels. To take an appropriate decision, the risk factors which can increase LBW incidence should be identified and well studied. To achieve this goal, there should be statistical methods that are flexible enough to address the data structure in a reasonable way. In this paper, we have applied a method that accommodates a scale effect which could occur during data aggregation from a lower to a higher geographical level. To this end, we found that income is negatively associated with LBW rate at both the county and PH levels. Further, we have shown that if the scaling effect is not properly handled, it can lead to erroneous conclusions. Hence, we recommend that the shared multiscale model should be used in practice for small area aggregated data. There are other complex multiscale models that can be used for aggregated data at different geographical levels [20]–[23]. The advantage of the shared multiscale model employed in this paper is that it can be easily implemented in standard software such as WinBUGS. Thus, it is very useful and valid for public health practitioners to adapt this method for similar problems that are encountered in practice.

Using multiscale model to handle the information at different scale levels is very important to answer different research questions: If some one is interested to study the relative risk at multiple scale levels, our multiscale model is useful to answer such research question. Further, the multiscale model can be used to estimate the relative risk at a single scale level. In particular, our method is useful to study whether or not a covariate effect at the finer level will hold true at the coarser level.

Although we have shown that income has a negative impact on LBW rate at both county and PH levels, further research is needed to investigate the validity of these results after including other important risk factors in the model. It is known that other risk factors such as smoking, poor nutrition, alcohol and drug use, race, and maternal stress are associated with LBW incidence. The effect of income on LBW incidence may be changed when we incorporate these risk factors to the multiscale model. The method which we applied here can be easily extended to include those risk factors. Therefore, further work should be done to assess whether or not income and other risk factors jointly affect LBW incidence.

Finally, we conclude that using the shared multiscale model is a useful and a novel method for public health application to get an accurate risk estimate that can be used for planning purpose.
