# Supplementary material for: Impact of Income on Small Area Low Birth Weight Incidence Using Multiscale Models
Source: AIMS Public Health. 2015 Oct 10;2(4):667–80. doi: 10.3934/publichealth.2015.4.667 (PMC4936536; doi:10.3934/publichealth.2015.4.667)
Supplement: Supplementary file 1 [file publichealth-02-04-667-s001.pdf]

10. Besag J, York J, Mollié A (1991) Bayesian image restoration with applications in spatial statistics (with discussion). *Ann Inst Stat Math*, 43:1-59.

11. Gelman A (2006) Prior distribution for variance parameters in hierarchical models. *Bayesian Analysis*, 3:515-533.

12. Spiegelhalter DJ, Best NG, Carlin BP et al. (2002) Bayesian measures of model complexity and fit. *J R Stat Soc Series B*, 64: 583-639.

13. Gelman A, Carlin JB, Stern HS et al. (2004) *Bayesian Data Analysis*. New York: Chapman and Hall.

14. Lawson AB (2013) *Bayesian Disease Mapping: Hierarchical Modeling in Spatial Epidemiology*. 2nd ed. New York: Chapman and Hall/CRC Press.

15. Gelman A, Rubin DB (1992) Inference from iterative simulation using multiple sequences (with discussion). *Stat Sci*, 7:457-511.

16. Mathews TJ, MacDorman MF (2013) Infant Mortality Statistics from the 2009 Period Linked Birth/Infant Death Data Set. *National Vital Statistics Reports*, 61(8).

17. Martin JA, Hamilton BE, Sutton PD, et al. (2009) Births: Final data for 2006. *National Vital Statistics Reports*, 57(7).

18. World Health Organization: Feto-maternal nutrition and low birth weight. Available from: http://www.who.int/nutrition/topics/feto-maternal/en/. Retrieved 2015-07-23.

19. Borders AE, Grobman WA, Amsden LB, et al. (2007) Chronic stress and low birth weight neonates in a low-income population of women. *Obstetrics and Gynecology*, 109:331-338.

20. Kolaczyk ED, Huang H (2001) Multiscale statistical models for hierarchical spatial aggregation. *Geogr Anal*, 33:95-118.

21. Louie MM, Kolaczyk ED (2006) A multiscale method for disease mapping in spatial epidemiology. *Stat Med*, 25:1287–1306.

22. Louie MM, Kolaczyk ED (2006) Multiscale detection of localized anomalous structure in aggregate disease incidence data. *Stat Med*, 25:787–810.

23. Louie MM, Kolaczyk ED (2004) On the covariance properties of certain multiscale spatial processes. *Statist Probab Lett*, 66:407–416.

Supplementary

A WinBUGS program invoked via the R package for Model 1 is as follows:

```
###Code for Model 1###############
model
  {
     ######### county level#########
      for (i in 1:m)
```

```
    {
     yc[i]~dbin(muc[i],nc[i])                        #likelihood
     muc[i]<-thc[i]                                  #Probability of LBW
     logit(thc[i])<-a0+v[i]+u[i]+beta1*(incomeS[i])#Convolution Model
     v[i]~dnorm(0,tauV)                              #Uncorrelated random effect
     ypred[i] ~ dbin(muc[i],nc[i])                   #Predicted Value
     PPL[i] <- pow(ypred[i]-yc[i],2)
 }
 ######### PH level#########
 for (j in 1:p)
    {
     yph[j]~dbin(mph[j],nph[j])                              #likelihood
     mph[j]<-thp[j]                                          #Probability of LBW
     logit(thp[j])<-a0ph+vph[j]+uph[j]+alpha1*(incomePHS[j])#Convolution Model
     vph[j]~dnorm(0,tauVPH)                                  #UH
     ypredph[j] ~dbin(mph[j],nph[j])                         #Predicted Value
     PPLph[j] <- pow(ypredph[j]-yph[j],2)
}
  #####County level####
for (k in 1: nsumc)
    {
     weic[k]<-1                                              #Weight for ICAR
}
  #####PH level####
for (k in 1 :nsumph)
    {
     weiph[k]<-1                                             #Weight for ICAR
}
   #####County level####
mspec <- mean(PPL[])                               #MSPE
u[1:m]~car.normal(adjc[],weic[],numc[],tauU)       #ICAR prior distribution
a0~dflat()                                         #Flat prior for the intercept
beta1~dnorm(0,taubeta1)                            #Normal prior for the slope
taubeta1<-pow(sdbeta1,-2)
sdbeta1~dunif(0,100)
tauV<-1/pow(sdV,2)
sdV~dunif(0,100)                                   #Uniform prior distribution
sdU~dunif(0,100)
tauU<-1/pow(sdU,2)
  #####PH level####
mspecph <- mean(PPLph[])                           #MSPE
uph[1:18]~car.normal(adjph[],weiph[],numph[],tauph)#ICAR prior distribution
a0ph~dflat()                                       #Flat prior for the intercept
```

```
    tauph<-1/pow(sdph,2)
    sdph~dunif(0,100)
    sdVPH~dunif(0,100)                                  #Uniform prior distribution
    tauVPH<-1/pow(sdVPH,2)
    alpha1~dnorm(0,taualpha1)                           #Normal prior for the slope
    taualpha1<-pow(sdalpha1,-2)
    sdalpha1~dunif(0,100)
 }
```

A WinBUGS program invoked via the R package for Model 2 is as follows:

```
###Code for Model 1###############
model
  {
     ######### county level#########
      for (i in 1:m)
          {
           yc[i]~dbin(muc[i],nc[i])                                        #likelihood
           muc[i]<-thc[i]                                                  #Probability of LBW
           logit(thc[i])<-a0+v[i]+u[i]+beta1*(incomeS[i])+uph[phc[i]#Convolution Model
           v[i]~dnorm(0,tauV)                                              #UH
           ypred[i] ~ dbin(muc[i],nc[i])                                   #Predicted Value
           PPL[i] <- pow(ypred[i]-yc[i],2)
      }
     ######### PH level#########
     for (j in 1:p)
         {
          yph[j]~dbin(mph[j],nph[j])                                       #likelihood
          mph[j]<-thp[j]                                                   #Probability of LBW
          logit(thp[j])<-a0ph+vph[j]+uph[j]+alpha1*(incomePHS[j])   #Convolution Model
          vph[j]~dnorm(0,tauVPH)                                           #UH
          ypredph[j] ~dbin(mph[j],nph[j])                                  #Predicted Value
          PPLph[j] <- pow(ypredph[j]-yph[j],2)
    }
      #####County level####
    for (k in 1: nsumc)
        {
         weic[k]<-1                                          #Weight for ICAR structure
    }
      #####PH level####
    for (k in 1 :nsumph)
        {
         weiph[k]<-1                                         #Weight for ICAR structure
    }
```

```
      #####County level####
    mspec <- mean(PPL[])                                #MSPE
    u[1:m]~car.normal(adjc[],weic[],numc[],tauU)     #ICAR prior distribution
    a0~dflat()                                          #Flat prior for the intercept
    beta1~dnorm(0,taubeta1)                             #Normal prior for the slope
    taubeta1<-pow(sdbeta1,-2)
    sdbeta1~dunif(0,100)
    tauV<-1/pow(sdV,2)
    sdV~dunif(0,100)                                    #Uniform prior distribution
    sdU~dunif(0,100)
    tauU<-1/pow(sdU,2)
      #####PH level####
    mspecph <- mean(PPLph[])                              #MSPE
    uph[1:18]~car.normal(adjph[],weiph[],numph[],tauph)#ICAR prior distribution
    a0ph~dflat()                                          #Flat prior for the intercept
    tauph<-1/pow(sdph,2)
    sdph~dunif(0,100)
    sdVPH~dunif(0,100)                                    #Uniform prior distribution
    tauVPH<-1/pow(sdVPH,2)
    alpha1~dnorm(0,taualpha1)                             #Normal prior for the slope
    taualpha1<-pow(sdalpha1,-2)
    sdalpha1~dunif(0,100)
 }
 #uph[phc[i] represents the shared correlated random effect,
 #i.e., the common random effect for the counties within a given PH district
 #phc is a vector of indices that represents
#which county belong to which public health district (PH)
#Note that we have 159 counties that are grouped into 18 PH districts.
#phc=c(18,18,18,3,17,10,5,9,7,7,17,13,18,7,1,18,16,15,3,1,18,12,9,18,1,
8,9,14,5,8,2,18,11,18,3,16,7,12,17,8,9,10,3,4,13,8,3,11,3,7,1,5,16,18,
14,15,9,10,10,6,14,16,1,9,3,5,12,10,10,17,9,8,10,15,15,17,7,5,17,18,16,
16,13,2,15,7,13,3,1,16,1,7,10,8,5,8,16,1,15,3,3,17,13,5,14,8,12,5,5,9,
17,14,18,15,9,13,17,8,10,8,16,12,8,16,3,15,10,8,8,8,16,18,8,13,3,3,7,
18,10,13,15,7,17,10,15,9,5,18,16,17,18,8,13,10,14,13,16,17,3)
```

AIMS Press

© 2015, Mehreteab, licensee AIMS Press. This is an open access article distributed under the terms of the Creative Commons Attribution License (http://creativecommons.org/licenses/by/4.0)
